# Effect of pregravid obesity on perinatal outcomes in singleton pregnancies following in vitro fertilization and the weight-loss goals to reduce the risks of poor pregnancy outcomes: A retrospective cohort study

**DOI:** 10.1371/journal.pone.0227766

**Published:** 2020-02-13

**Authors:** Lu Liu, Hongmei Wang, Yang Zhang, Jinlei Niu, Zhongyuan Li, Rong Tang

**Affiliations:** 1 Reproductive Hospital Affiliated to Shandong University, Jinan, China; 2 International Peace Maternity & Child Health Hospital Affiliated to Shanghai Jiao Tong University, Shanghai, China; 3 Department of Obstetrics & Gynecology, Provincial Hospital Affiliated to Shandong University, Jinan, China; 4 National Research Center for Assisted Reproductive Technology and Reproductive Genetics, Jinan, China; 5 The Key Laboratory of Reproductive Endocrinology (Shandong University), Ministry of Education, Jinan, China; 6 Shandong Provincial Key Laboratory of Reproductive Medicine, Jinan, China; University of Insubria, ITALY

## Abstract

**Objective:**

In the present study, we aimed to determine whether pregravid obesity independently predicts increased risks of perinatal complications following in vitro fertilization (IVF) and the weight loss goals to reduce the risk of poor pregnancy outcomes.

**Design:**

Retrospective cohort study.

**Population:**

All pregnancies after first the fresh IVF cycle from January 2014 to December 2016 in the Reproductive Center affiliated to Shandong University were reviewed. A total of 3,962 eligible singleton births were stratified into cohorts based on the body mass index (BMI) definitions of the Working Group on Obesity in China (WGOC).

**Main outcome measures:**

Adverse perinatal outcomes.

**Results:**

Pregravid overweight and obesity were associated with increased risks of gestational diabetes mellitus (GDM), hypertensive disorders of pregnancy (HDP), including gestational hypertension (GH) and pre-eclampsia (PE), polyhydramnios, preterm premature rupture of the membranes (PPROM), placental abruption, preterm birth (PTB) <37 weeks, caesarean section (CS), fetal macrosomia, large for gestational age (LGA) >90th percentile, neonatal respiratory distress syndrome (NRDS), neonatal intensive care unit (NICU) admission and congenital anomalies as compared with the normal-weight group after adjustment of differences in age, parity, polycystic ovary syndrome (PCOS) and type of controlled ovarian hyperstimulation (COH). The increased risks of PPROM, NRDS and congenital anomalies were eliminated after adjustment of GDM development, whereas the increased risk of NRDS disappeared after adjustment of HDP. Placenta previa was not significantly different between the obese group and reference group (REF). Moreover, the rates of postpartum hemorrhage (PPH), PTB<32 weeks, small for gestational age (SGA) >90th percentile and perinatal mortality were also not significantly different between above-mentioned two groups. For obese women, a 10%-15% reduction in prepregnancy BMI was associated with significantly decreased risks of GH, CS and fetal macrosomia. For overweight women, just a 5% reduction in BMI could significantly reduce the risks of GDM, CS and fetal macrosomia.

**Conclusions:**

Pregravid obesity could independently predict a higher risk of adverse pregnancy outcomes after adjustment of differences in maternal age, parity, PCOS, and type of COH in IVF pregnancies. The potential mechanism that obesity potentiated the risks of some poor perinantal outcomes might occur through the development of GDM and HDP. A 10%-15% reduction in pregravid BMI for obese women and a 5% reduction for overweight women were associated with a significant reduction of poor perinatal complications.

## Introduction

Obesity is a major global health issue, and its severity is increasing in recent years. The worldwide proportion of women with a body mass index (BMI) of above 25 kg/m^2^ has increased from 29.8% in 1980 to 38% in 2013, which is largely driven by new cases from Asia [1]. In Asia, the prevalence of obesity is very low previously, while it is increasing at an alarming rate recently, especially in China, Japan and India [2]. The number of Chinese obese people is below 0.1 million in 1975, while such number has reached 43.2 million in 2014, accounting for 16.3% of worldwide obesity [3]. As obesity and overweight have become one of the most important threats to human health in general, it has also become one of the most common medical conditions complicating pregnancies of women of reproductive age. Now it is not uncommon for overweight and obese women to seek fertility treatment, such as in vitro fertilization (IVF) [4]. Previous studies have found that the presence of excessive maternal adipose tissue is linked to a number of important adverse outcomes in spontaneous pregnancies. However, the effects of obesity on risks of maternal and fetal adverse outcomes in pregnancies following successful IVF remain largely unexplored.

The 2013 American College of Obstetricians and Gynecologists strongly recommends preconception counseling for overweight and obese women about maternal and fetal risks in pregnancy and encourage them to undertake a weight-loss program [5]. Until now, there is insufficient data regarding the effects of weight loss on the risks of perinatal complications. The gold standard evidence to inform this counseling would come from randomized trials of preconceptional weight-loss interventions. However, such studies are difficult to conduct in IVF pregnancies. Therefore, population-based studies comparing the pregnancy outcomes of different women based on their pre-IVF BMI are important to provide weight-loss goals prior to conception with the aim to reduce perinatal complications.

The aim of this study was to evaluate whether pre-IVF obesity independently predicts increased pregnancy complications after adjusting for important confounders. We also aimed to provide recommendations for Chinese women about the magnitude of weight loss prior to IVF for better perinatal outcomes.

## Materials and methods

### Study design

**Fig 1:**
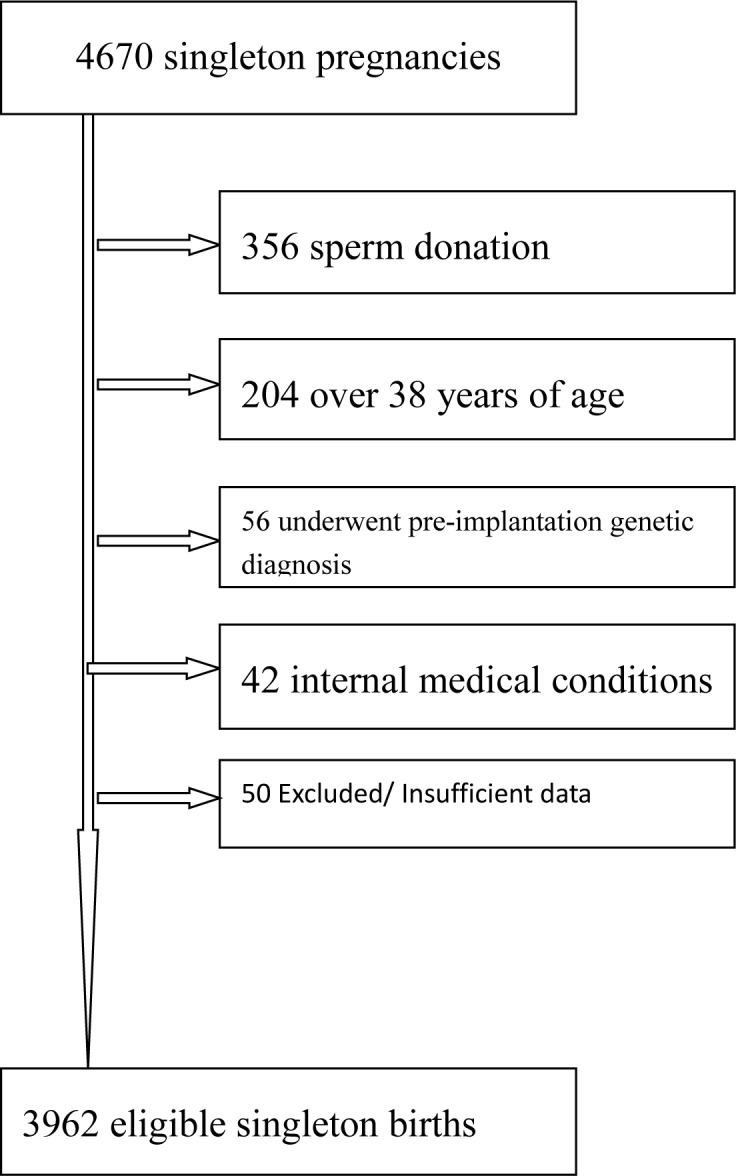
Flow chart depicting outcome of all singleton pregnancies screened.

This retrospective cohort study was carried out at the Reproductive Medical Center affiliated to Shandong University. The Centre routinely collects pregnancy and delivery information from postpartum patients. Women who underwent their first IVF cycle and delivered a single live infant (vanishing twin and selective reduction were excluded) at ≥28 weeks of gestation were enrolled in the cohort. Those who had internal medical conditions, especially pre-IVF hypertension and mellitus diabetes, recurrent spontaneous abortion (defined as three or more previous spontaneous miscarriages), cervical incompetence or chromosomal abnormality were excluded from the present study. To eliminate age as an independent variable for IVF pregnancy, women aged 38 years or older were excluded from this study. Of the 4,670 charts identified with a singleton live birth, 356 subjects used donor sperm, 204 women were over 38 years of age, 42 women had internal medical conditions, 56 cases had chromosomal abnormality and underwent preimplantation genetic diagnosis, and 50 births did not meet inclusion criteria or contained insufficient information The flow chart was presented in Fig 1.

Eventually, a total of 3,962 women were included in the final analysis. Of these enrolled women, 584 subjects had polycystic ovary syndrome (PCOS), and 3,378 women did not. They were categorized into three groups according to their BMI, which was measured at the initial IVF consultation (weight [kilograms]/height [meters]^2^). The WHO expert consultation has reviewed scientific evidence and suggested that Asian populations have different associations between BMI, percentage of body fat, and health risks compared with European populations. They conclude that the proportion of Asian people with a high risk of type 2 diabetes and cardiovascular disease is substantial at BMI lower than the existing WHO cut-off point for overweight (> or = 25 kg/m^2^) [6]. Since only Asian women were included in the present study, it might be more reasonable to classify them according to the BMI definitions of the Working Group on Obesity in China (WGOC). BMI groups were defined as follows: normal weight (BMI<24.00 kg/m^2^), overweight (BMI 24.00–27.99 kg/m^2^) and obese (BMI≥28.00 kg/m^2^).

Most of the studies on effects of BMI on perinatal outcomes have focused on spontaneous pregnancies. Therefore, PCOS is often a confounding factor being omitted. However, it remains unclear whether the reported effects of obesity on pregnancy outcome are independent of the effects of PCOS. We therefore performed two subgroup analyses to isolate the effect of obesity from PCOS on pregnancy outcomes.

For those outcomes significantly associated with pregravid obesity or overweight, additional analyses were carried out to compare the target BMI group and corresponding BMI reduction group. Because the National Institutes of Health (NIH) recommends a 10% reduction in body weight to confer health benefits outside of pregnancy [7], a 10% reduction in prepregnancy BMI was defined as the BMI reduction goal to make the weight-loss model. For example, the risks among women with a prepregnancy BMI of 30–32 were compared with risks among women with a BMI of 27–29, which represents approximately a 10% reduction in BMI. Four models were conducted as follows: group with a BMI of 30–32 vs. group with a BMI of 27–29; group with a BMI of 28–29 vs. group with a BMI of 25–26; group with a BMI of 26–27 vs. group with a BMI of 23–24; group with a BMI of 24–25 vs. group with a BMI of 21–22, and the BMI reduction group was approximately a 10% reduction of the target BMI group. Logistic regressions were performed between the target BMI group and BMI reduction group (the control group) separately. If there was a statistically significant increase in the risks of poor pregnancy outcomes between the target BMI group and BMI reduction group, the target BMI group was continuously compared with a smaller BMI reduction group, which presents only a 5% difference in prepregnancy BMI. For example, group with a BMI of 26–27 vs. group with a BMI of 24–25; group with a BMI of 24–25 vs. group with a BMI of 22–23; the BMI reduction group was approximately a 5% reduction of the target BMI group. On the contrary, if there was no statistically significant difference between the target BMI group and the control group, a stricter group which presents a greater magnitude of weight loss (a 15% difference in pre-IVF BMI) was defined as the control group. For example, group with a BMI of 30–32 vs. group with a BMI of 25–27; group with a BMI of 28–29 vs. group with a BMI of 24–25.

### Outcomes

The following adverse maternal and perinatal outcomes were examined: 1) gestational diabetes mellitus (GDM) was diagnosed via the oral glucose tolerance test (75 g, 2 h) [8], 2) hypertensive disorders of pregnancy (HDP), including gestational hypertension (GH) and pre-eclampsia (PE) as per the International Society for the Study of Hypertension in Pregnancy guidelines [9], 3) polyhydramnios was defined as amniotic fluid index (AFI) >24 cm, whereas oligohydramnios was defined as AFI <8 cm, 4) placenta previa (PP) refers to that the placenta partially or completely obstructs the internal orifice of the cervix by lying the lower uterine segment, 5) placental abruption was defined as the premature detachment of the placenta from the uterine wall before birth and after 20 weeks’ gestation, 5) postpartum hemorrhage (PPH) was defined as blood loss of more than 500 mL within 24 h after vaginal delivery or more than 1,000 mL after caesarean section (CS), 6) PPROM, and 7) mode of delivery (rate of CS).

Birth outcome variables included gestational age (GA) at delivery (week), birth weight (g), birth height (cm), preterm birth (PTB)<32 and <37 weeks, low birth weight (LBW<1,500 and <2,500 g), macrosomia (>4,000 g), small for gestational age and large for gestational age (SGA and LGA; <10th and >90th percentiles, respectively, according to Fenton 2013 growth curves [10]), neonatal respiratory distress syndrome (NRDS) (defined as one or more signs of increased work of breathing, such as tachypnea, nasal flaring, chest retractions and grunting), congenital malformations, and perinatal mortality (≤28 days).

### Statistical analysis

Statistical analysis was performed with SPSS 20.0. Descriptive statistical methods were used to summarize the study population. Participant characteristics were summarized using median and interquartile range (IQR) for continuous variables, and counted with percentages (%) for categorical variables. The Wilcoxon rank-sum test was used to evaluate differences between continuous variables, and Fisher’s exact test and X^2^ were performed for categorical variables to compare data of the three BMI categories. For each outcome, logistic regression was used to estimate odds ratio (OR) and 95% confidence interval (CI). Initially, unadjusted ORs were calculated for all outcomes by fitting univariable logistic regression models. Then, multiple logistic regression models were constructed to examine the magnitude and significance of the independent effect of BMI by adjusting maternal age, parity, PCOS, and type of controlled ovarian hyperstimulation (COH). To demonstrate how obesity affected neonatal outcomes, PTB was also adjusted (in addition to age, parity, PCOS and type of COH) in logistic regression analyses. A P value of <0.05 was considered as statistically significant.

## Results

### Population characteristics

A total of 3,962 singleton births were assessed for selected adverse pregnancy and birth outcomes. Table 1 lists the baseline characteristics of all participants. The obese population was significantly older than the normal-weight population and less frequently diagnosed with tubal factor. The proportion of women with PCOS in the obese population was significantly higher compared with the normal-weight group. The rate of “long agonist protocol” used in COH was significantly lower in overweight women compared with the other two groups. Parity was not significantly different across groups.

**Table 1:** Baseline characteristics of women achieving singleton pregnancies by BMI category.

Parameter	<24.00	24.00–27.99	≥28.00	P value
	(n = 2,485)	(n = 1,033)	(n = 444)	
Female age(years)	29(27–32)	31(27–34)	30(27–33)	**<0.001^a^^.^^b^**
Cause for infertility(%)				
Male factor	365/2485(14.69)	135/1033(13.07)	48/444 (10.82)	0.066
Tubal factor	1411/2485(56.78)	555/1033(53.73)	193/444(43.47	**<0.001^b^^.^^c^**
Ovulatory disorder (PCOS)	255/2485(10.26)	179/1033(17.33)	150/444(33.78)	**<0.001^a^^.^^b^^.^^c^**
Endometriosis	158/2485(6.36)	51/1033(4.94)	18/444(4.05)	0.070
Unexplained infertility	150/2485(6.04)	61/1033(5.91)	15/444(3.38)	0.080
Other	146/2485(5.88)	52/1033(5.03)	20/444(4.50)	0.160
Parity				0.329
Primiparous	1341/2485(53.96)	533/1033(51.60)	199/444(44.82)	
Multiparous	1144/2485(46.04)	500/1033(38.46)	245/444(55.18)	
Type of COH				
Long agonist	1607/2485(64.67)	630/1033(60.99)	301/444(67.79)	**0.027^a^^.^^c^**
Short agonist	518/2485(20.85)	253/1033(24.49)	79/444(17.79)	**0.008^a^^.^^c^**
Antagonist	331/2485(13.32)	135/1033(13.07)	58/444(13.06)	0.975
Other	29/2485(1.17)	15/1033(1.45)	6/444(1.35)	0.776

Data were presented as median and interquartile range (IQR) or n (%).

a. Pairwise comparisons revealed a statistically significant difference between the first and second BMI categories.

b. Pairwise comparisons revealed a statistically significant difference between the first and third BMI categories.

c. Pairwise comparisons revealed a statistically significant difference between the second and third BMI categories.

### Perinatal outcomes

**Fig 2:**
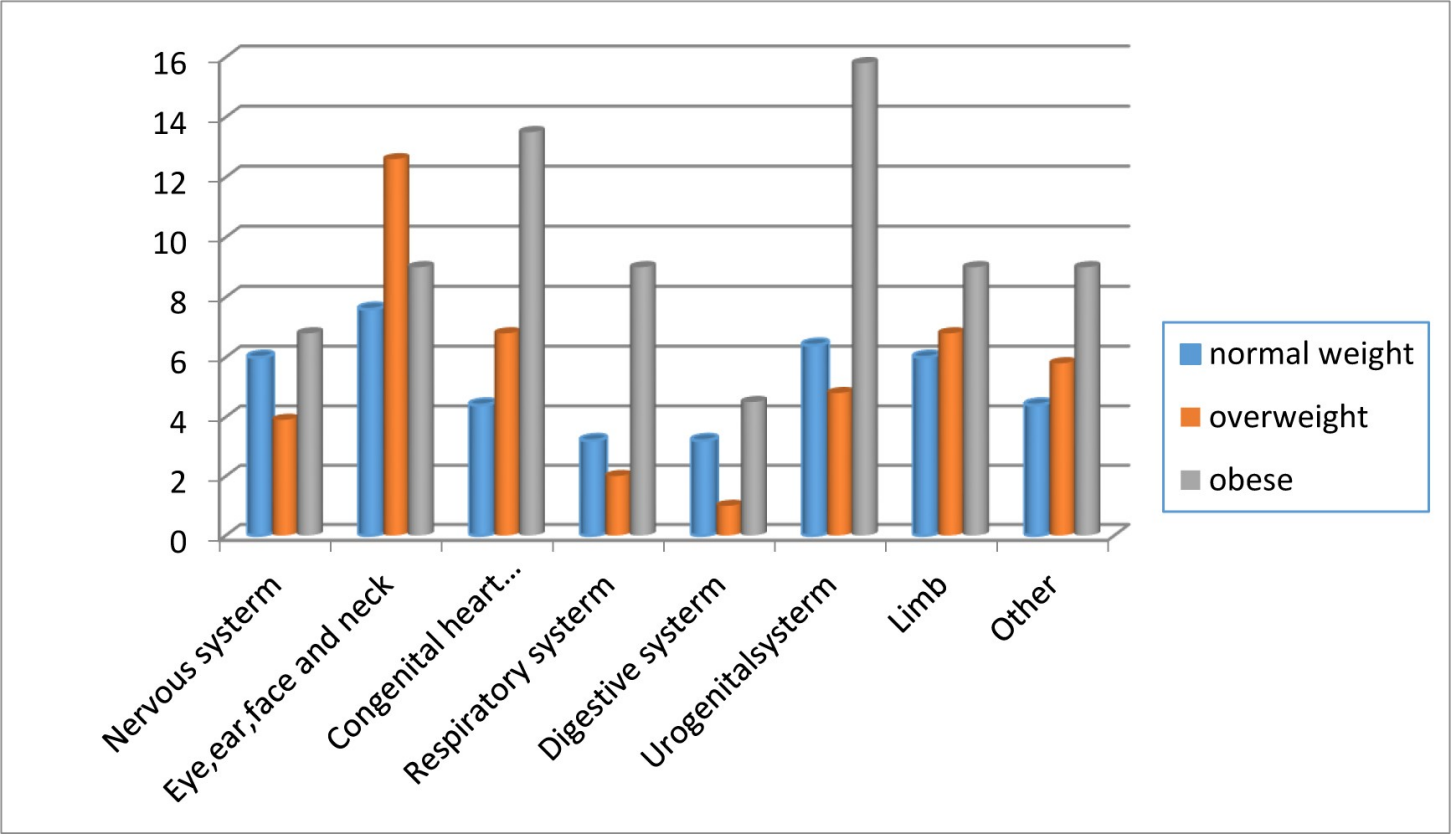
Prevalence of major congenital malformations in live singleton births conceived by IVF.

In the unadjusted analyses (Table 2), obesity was associated with increased risks of GDM, HDP, PPROM, placental abruption, PTB <37 weeks, CS, fetal macrosomia, LGA, LBW<2,500 g, NRDS, neonatal intensive care unit (NICU) admission and congenital anomalies. In the adjusted analyses (Table 3), the significantly increased risk of LBW<2,500 g disappeared, whereas the following pregnancy complications remained significant after adjustment of age, parity, PCOS, and type of COH: GDM (aOR: 2.32, 95% CI: 1.58–3.40), GH (aOR: 3.08, 95% CI: 2.11–4.50), PE (aOR: 2.92, 95% CI: 1.19–7.20), polyhydramnios (aOR: 2.25, 95% CI: 1.14–4.47), PPROM (aOR: 2.92, 95% CI: 0.94–2.77), placental abruption (aOR: 4.51, 95% CI: 1.30–15.60), PTB <37 weeks (aOR: 1.68, 95% CI: 1.18–2.37), CS (aOR: 2.19, 95% CI: 1.63–2.95), fetal macrosomia (aOR: 2.19, 95% CI: 1.63–2.95), NRDS (aOR: 3.17, 95% CI: 1.23–8.19), LGA (aOR: 2.33, 95% CI: 1.85–2.94), NICU admission (aOR: 1.51, 95% CI: 1.04–2.29) and congenital anomalies (aOR: 1.63, 95% CI: 1.04–2.56). The risks of GDM, CS, LGA, fetal macrosomia and NICU admission were considerably increased in the overweight and obese women compared with the normal-weight ones, whereas the remaining selected adverse pregnancy and birth outcomes appeared to be significantly increased only in the obese women. In the subgroup analysis of organ specific malformations, there was a statistically significant increase for malformations of the urogenital system and congenital heart defects in the obese population. Compared with offspring of normal-weight mothers, the aOR for urogenital system malformations was 2.48 (95% CI: 1.13–7.14) for obese mothers, and that for congenital heart defects was 2.30 (95% CI: 0.64–8.27). The results were presented in Fig 2.

**Table 2:** Unadjusted association between BMI and adverse perinatal outcomes.

Parameter	<18.50 (REF)	18.50–24.99	OR (95%CI)	P value	≥25.00	OR (95%CI)	P value
	(n = 2485)	(n = 1033)			(n = 444)		
GDM(%)	99/2485(3.98)	83/1033(8.03)	2.11(1.56–2.85)	**<0.001**	44/444(9.91)	2.65(1.83–3.84)	**<0.001**
HDP(%)	84/2485(3.38)	47/1033(4.55)	1.36(0.95–1.96)	0.096	51/444(11.49)	3.71(2.58–5.34)	**<0.001**
Preeclampsia(%)	13/2485(0.52)	7/1033(0.68)	1.30(0.52–3.26)	0.58	9/444(2.03)	3.93(1.67–9.26)	**0.002**
Polydydramnios(%)	29/2485(1.17)	21/1033(2.03)	1.76(1.00–3.10)	0.051	13/444(2.93)	2.55(1.32–4.95)	**0.006**
Oligodydramnios(%)	144/2485(5.79)	59/1033(5.71)	0.99(0.72–1.35)	0.923	24/444(5.41)	0.93(0.60–1.45)	0.745
PPPOM(%	34/2485(1.37)	23/1033(2.23)	1.64(0.96–2.80)	0.069	12/444(2.70)	2.00(1.03–3.90)	**0.041**
PP(%)	85/2485(3.42)	27/1033(2.61)	0.76(0.49–1.18)	0.216	12/444(2.70)	0.78(0.43–1.45)	0.437
Placental abruption(%)	76/2485(3.06)	5/1033(0.48)	2.01(0.61–6.60)	0.25	5/444(1.13)	4.71(1.43–15.49)	**0.011**
PTB<32weeks(%)	24/2485(0.97)	9/1033(0.87)	0.90(0.42–1.95)	0.791	7/444(1.58)	1.64(0.70–3.84)	0.251
PTB<37weeks (%)	159/2485(6.40)	80/1033(7.74)	1.23(0.93–1.62)	0.149	49/444(11.04)	1.82(1.30–2.54)	**0.001**
CS(%)	1889/2485(76.02)	866/1033(83.83)	1.63(1.35–1.97)	**<0.001**	385/444(86.71)	2.06(1.54–2.74)	**<0.001**
PPH(%)	36/2485(1.45)	14/1033(1.36)	0.94(0.50–1.74)	0.831	3/444(0.68)	0.46(0.14–1.51)	0.201
Fetal macrosomia(%)	309/2485(12.43)	198/1033(19.17)	1.67(1.37–2.03)	**0.001**	105/444(23.65)	2.18(1.70–2.80)	**<0.001**
Respiratory distress(%)	12/2485(0.48)	12/1033(1.16)	1.81(0.76–4.31)	0.179	7/444(1.54)	3.30(1.29–8.43)	**0.013**
SGA(%)	97/2485(3.90)	24/1033(2.32)	0.59(0.37–0.92)	**0.021**	13/444(2.93)	0.74(0.41–1.34)	0.321
LGA(%)	432/2485(17.38)	282/1033(27.30)	1.78(1.50–2.12)	**<0.001**	146/444(32.88)	2.33(1.86–2.91)	**<0.001**
LBW<1,500g(%)	8/2485(0.32)	5/1033(0.48)	1.51(0.49–4.61)	0.474	5/444(1.13)	3.53(1.15–10.83)	**0.028**
LBW<2,500g(%)	91/2485(3.66)	33/1033(3.19)	0.87(0.58–1.30)	0.494	18/444(4.05)	1.11(0.66–1.86)	0.688
GA(w)	39.2(38.3–40.0)	39.1(38.2–40.0)	N#A	0.701	39.0(38–39.6)	N#A	**<0.001**
BW(g)	3400(3150–3700)	3560(3250–3893)	N#A	**<0.001**	3600(3200–3950)	N#A	**<0.001**
BH(cm)	50(50–51)	50(50–51)	N#A	0.156	50(50–51)	N#A	**0.004**
NICU admission(%)	156/2485(6.28)	94/1033(9.10)	1.50(1.14–1.95)	**0.003**	52/444(11.71)	1.98(1.42–2.76)	**<0.001**
Congenital anomalies(%)	94/2485(3.78)	41/1033(3.97)	1.05(0.72–1.53)	0.793	29/444(6.53)	1.78(1.16–2.73)	**0.009**
Mortality(%)	12/2485(0.48)	5/1033(0.48)	1.00(0.35–2.85)	0.996	3/444(0.68)	1.40(0.39–4.99	0.602

**Table 3:** Adjusted association between BMI and adverse perinatal outcomes. (adjusted for age, PCOS, parity and type of COH).

Parameter	<18.50 (REF)	18.50–24.99	OR (95%CI)	P value	≥25.00	OR (95%CI)	P value
	(n = 2485)	(n = 1033)			(n = 444)		
GDM(%)	99/2485(3.98)	83/1033(8.03)	2.05(1.51–2.78)	**<0.001**	44/444(9.91)	2.32(1.58–3.40)	**<0.001**
HDP(%)	84/2485(3.38)	47/1033(4.55)	1.24(0.86–1.79)	0.256	51/444(11.49)	3.08(2.11–4.50)	**<0.001**
Preeclampsia(%)	13/2485(0.52)	7/1033(0.68)	1.18(0.47–3.00)	0.725	9/444(2.03)	2.92(1.19–7.20)	**0.020**
Polydydramnios(%)	29/2485(1.17)	21/1033(2.03)	1.62(0.91–2.87)	0.102	13/444(2.93)	2.25(1.14–4.47)	**0.020**
Oligodydramnios(%)	144/2485(5.79)	59/1033(5.71)	0.99(0.72–1.35)	0.941	24/444(5.41)	0.95(0.60–1.49)	0.809
PPPOM(%	34/2485(1.37)	23/1033(2.23)	1.62(0.94–2.77)	0.082	12/444(2.70)	1.62(0.94–2.77)	**0.048**
PP(%)	85/2485(3.42)	27/1033(2.61)	0.78(0.50–1.21)	0.267	12/444(2.70)	0.90(0.48–1.67)	0.731
Placental abruption(%)	76/2485(3.06)	5/1033(0.48)	2.06(0.62–6.84)	0.238	5/444(1.13)	4.51(1.30–15.60)	**0.017**
PTB<32weeks(%)	24/2485(0.97)	9/1033(0.87)	0.90(0.41–1.95)	0.784	7/444(1.58)	1.39(0.57–3.37)	0.464
PTB<37weeks (%)	159/2485(6.40)	80/1033(7.74)	1.19(0.90–1.58)	0.220	49/444(11.04)	1.68(1.18–2.37)	**0.004**
CS(%)	1889/2485(76.02)	866/1033(83.83)	1.65(1.36–2.00)	**<0.001**	385/444(86.71)	2.19(1.63–2.95)	**<0.001**
PPH(%)	36/2485(1.45)	14/1033(1.36)	1.01(0.54–1.89)	0.973	3/444(0.68)	0.56(0.17–1.86)	0.346
Fetal macrosomia(%)	309/2485(12.43)	198/1033(19.17)	1.69(1.39–2.06)	**<0.001**	105/444(23.65)	2.28(1.76–2.95)	**<0.001**
Respiratory distress(%)	12/2485(0.48)	12/1033(1.16)	1.73(0.72–4.19)	0.222	7/444(1.54)	3.17(1.23–8.19)	**0.017**
SGA(%)	97/2485(3.90)	24/1033(2.32)	0.62(0.39–0.99)	**0.038**	13/444(2.93)	0.84(0.46–1.54)	0.578
LGA(%)	432/2485(17.38)	282/1033(27.30)	1.77(1.49–2.11)	<0.001	146/444(32.88)	2.33(1.85–2.94)	**<0.001**
LBW<1,500g(%)	8/2485(0.32)	5/1033(0.48)	1.24(0.39–4.00)	0.718	5/444(1.13)	1.90(0.56–6.49)	0.306
LBW<2,500g(%)	91/2485(3.66)	33/1033(3.19)	0.71(0.44–1.15)	0.160	18/444(4.05)	0.64(0.34–1.19)	0.155
NICU admission(%)	156/2485(6.28)	94/1033(9.10)	1.40(1.05–1.88)	**0.022**	52/444(11.71)	1.51(1.04–2.29)	**0.032**
Congenital anomalies(%)	94/2485(3.78)	41/1033(3.97)	1.03(0.71–1.51)	0.869	29/444(6.53)	1.63(1.04–2.56)	**0.032**
Mortality(%)	12/2485(0.48)	5/1033(0.48)	1.01(0.35–2.94)	0.980	3/444(0.68)	1.31(0.35–4.88)	0.692

PTB<37 weeks and PPH seemed to have a less significant association with BMI in the present study. We observed that obese women had a slightly higher rate of LBW<2,500g, but a lower rate of LBW<1,500 g. Even though both of them were not statistically significant between the obese group and reference group (REF), GA at birth was significantly lower in obese pregnancies not only compared with the normal-weight group but also the overweight group. There were no significant differences in rates of SGA, PP or perinatal mortality.

To assess whether the increased risk of perinatal complications might be mediated by development of GDM and HDP, we performed logistic regressions for those outcomes with a significant association with pre-IVF BMI that was adjusted for development of GDM and HDP separately (in addition to age, PCOS, parity and type of COH) (Table 4). There were no longer increased risks of PPROM (aOR: 1.94, 95% CI: 0.97–3.85, P = 0.060), NRDS (aOR: 2.59, 95% CI: 0.96–7.01, P = 0.061) and congenital anomalies (aOR: 1.54, 95% CI: 0.98–2.43, P = 0.061) once the development of GDM for obese women was adjusted when compared with the normal-weight women. The increased risk of NRDS was eliminated after adjustment of HDP development (aOR: 2.58, 95% CI: 0.95–7.01, P = 0.063).

**Table 4:** Perinatal outcomes by BMI category when GDM/HDOP was adjusted (in addition to age, PCOS, parity and type of COH).

Adjusted for	Age, PCOS, parity, type of COH,GDM		Age, PCOS, parity, type of COH,GDM	
	aOR(95%CI)	P value	aOR(95%CI)	P value
GDM				
overweight	N/A	N/A	2.03(1.49–2.75)	**<0.001**
obese	N/A	N/A	2.11(1.43–3.12)	**<0.001**
HDP				
overweight	1.18(0.81–1.71)	0.390	N/A	N/A
obese	2.84(1.93–4.17)	**<0.001**	N/A	N/A
Preeclampsia				
overweight	1.13(0.44–2.89)	0.794	N/A	N/A
obese	2.75(1.10–6.88)	**0.030**	N/A	N/A
Polyhydramnios				
overweight	1.49(0.83–2.66)	0.178	1.62(0.91–2.87)	0.101
obese	2.05(1.03–4.11)	**0.042**	2.30(1.16–4.56)	**0.017**
PPROM				
overweight	1.57(0.92–2.71)	0.102	1.62(0.94–2.78)	0.080
obese	1.94(0.97–3.85)	0.060	2.08(1.05–4.13)	**0.037**
Placental abruption				
overweight	1.97(0.59–6.59)	0.270	2.04(0.61–6.77)	0.245
obese	4.40(1.26–15.39)	**0.020**	4.24(1.21–14.90)	**0.024**
PTB<37weeks				
overweight	1.16(0.87–1.53)	0.320	1.18(0.89–1.56)	0.257
obese	1.61(1.13–2.28)	**0.008**	1.49(1.05–2.13)	**0.027**
CS				
overweight	1.64(1.34–2.00)	**<0.001**	1.67(1.36–2.05)	**<0.001**
obese	2.12(1.55–2.89)	**<0.001**	1.98(1.45–2.71)	**<0.001**
Fetal macrosomia				
overweight	1.65(1.35–2.02)	**<0.001**	1.66(1.36–2.03)	**<0.001**
obese	2.21(1.71–2.87)	**<0.001**	2.22(1.70–2.89)	**<0.001**
NRDS				
overweight	1.65(0.68–4.02)	0.269	1.73(0.71–4.17)	0.226
obese	2.59(0.96–7.01)	0.061	2.58(0.95–7.01)	0.063
SGA				
overweight	0.62(0.40–0.98)	**0.040**	0.62(0.39–0.98)	**0.040**
obese	0.85(0.47–1.55)	0.591	0.82(0.45–1.50)	0.518
LGA				
overweight	1.73(1.46–2.06)	**<0.001**	1.75(1.47–2.09)	**<0.001**
obese	2.27(1.80–2.86)	**<0.001**	2.27(1.80–2.87)	**<0.001**
NICU admission				
overweight	1.38(1.03–1.84)	**0.031**	1.41(1.06–1.89)	**0.019**
obese	1.48(1.01–2.15)	**0.043**	1.52(1.04–2.22)	**0.029**
Congenital anomalies				
overweight	0.99(0.68–1.44)	0.945	1.04(0.71–1.51)	0.857
obese	1.54(0.98–2.43)	0.061	1.68(1.07–2.64)	**0.024**

### Subgroup analysis

#### Non-PCOS subgroup

In the group of women without PCOS, GDM, GH, PE, polyhydramnios, placental abruption, CS, fetal macrosomia, LGA, NRDS and NICU admission were significantly more common in the obese group compared with the normal-weight group (Table 5). However, the increased risks of PTB <37 weeks and placental abruption were no longer observed after adjustment of HDP.

**Table 5:** Obstetric and neonatal outcomes by BMI category in women without PCOS.

Parameter	<18.50 (REF)	18.50–24.99	OR (95%CI)	P value	≥25.00	OR (95%CI)	P value
	(n = 2230)	(n = 854)			(n = 294)		
GDM(%)	84/2230(3.77)	64/854(7.49)	2.10(1.50–2.94)	**0.001**	20/294(6.80)	1.89(1.14–3.13)	**0.014**
HDP(%)	71/2230(3.18)	31/854(3.63)	1.09(0.70–1.67)	0.711	27/294(9.18)	2.93(1.84–4.66)	**0.000**
Preeclampsia(%)	8/2230(0.36)	5/854(0.59)	1.68(0.54–5.17)	0.370	5/294(1.70)	4.91(1.59–15.21)	**0.006**
Polydydramnios(%)	24/2230(1.09)	19/854(5.71)	1.95(1.06–3,60)	**0.033**	6/294(5.88)	1.79(0.72–4.43)	0.209
Oligodydramnios(%)	130/2230(5.74)	46/854(4.95)	0.93(0.66–1.32)	0.695	20/294(7.00)	1.20(0.73–1.95)	0.475
PPPOM(%	32/2230(1.43)	20/854(2.34)	1.64(0.93–2.90)	0.087	6/294(2.04)	1.43(0.59–3.45)	0.430
PP(%)	80/2230(3.59)	23/854(2.69)	0.72(0.45–1.16)	0.180	12/294(4.08)	1.11(0.60–2.07)	0.734
Placental abruption(%)	5/2230(0.22)	4/854(0.47)	2.05(0.54–7.73)	0.296	3/294(1.02)	4.49(1.06–19.06)	**0.042**
PTB<32weeks(%)	20/2230(0.90)	5/854(0.59)	0.70(0.26–1.89)	0.483	4/294(1.36)	1.64(0.55–4.84)	0.373
PTB<37weeks (%)	139/2230(6.23)	63/854(7.38)	1.18(0.86–1.61)	0.305	29/294(9.86)	1.65(1.08–2.51)	**0.020**
CS(%)	1707/2230(76.55)	722/854(84.54)	1.12(0.38–3.29)	0.835	256/294(87.07)	3.55(1.27–9.92)	**0.016**
PPH(%)	36/2230(1.48)	13//854(1.52)	0.99(0.52–1.88)	0.960	1/294(0.34)	0.22(0.03–1.59)	0.133
Fetal macrosomia(%)	279/2230(12.32)	165/854(18.57)	1.09(1.37–2.09)	**<0.001**	64/294(22.69)	2.03(1.49–2.76)	**<0.001**
Respiratory distress(%)	11/2230(0.49)	5/854(0.59)	1.11(0.38–3.25)	0.224	6/294(2.04)	3.53(1.26–9.86)	**0.016**
SGA(%)	89/2230(3.99)	24/854(2.81)	0.71(0.45–1.12)	0.138	9/294(3.06)	0.77(0.38–1.55)	0.465
LGA(%)	387/2230(17.35)	235/854(27.52)	1.79(1.48–2.16)	<0.001	97/294(32.99)	2.33(1.78–3.04)	**<0.001**
LBW<1,500g(%)	7/2230(0.31)	3/854(0.35)	0.99(0.25–3.97)	0.642	2/294(0.68)	1.47(0.29–7.50)	0.642
LBW<2,500g(%)	81/2230(3.63)	23/854(2.69)	0.60(0.35–1.04)	0.069	11/294(3.74)	0.68(0.32–1.43)	0.309
GA(w)	39.2(38.3–40.0)	39.2(38.3–40.0)	N#A	**0.694**	39(38–39.6)	N#A	**0.001**
BW(g)	3400(3150–3700)	3550(3250–3900)	N#A	**<0.001**	3600(3200–3900)	N#A	**<0.001**
BH(cm)	50(50–50)	50(50–51)	N#A	**0.001**	50(50–51)	N#A	0.709
NICU admission(%)	133/2230(5.66)	66/854(8.13)	1.31(0.94–1.81)	0.112	31/294(12.32)	1.68(1.08–2.64)	**0.023**
Congenital anomalies(%)	84/2230(3.77)	34/854(3.98)	1.05(0.69–1.58)	0.829	18/294(6.12)	1.60(0.94–2.71)	0.085
Mortality(%)	11/2230(0.49)	5/854(0.59)	1.16(0.40–3.40)	0.787	2/294(0.68)	1.17(0.25–5.43)	0.838

#### PCOS subgroup

In patients with PCOS, GDM, HDP, PPROM, CS, fetal macrosomia and LGA were the outcomes that were significantly changed with the increase of BMI (Table 6).

**Table 6:** Obstetric and neonatal outcomes by BMI category in women with PCOS.

Parameter	<18.50 (REF)	18.50–24.99	OR (95%CI)	P value	≥25.00	OR (95%CI)	P value
	(n = 255)	(n = 179)			(n = 150)		
GDM(%)	15/255(5.88)	19/179(10.61)	1.95(0.96–3.95)	0.065	24/150(16.00)	3.07(1.55–6.06)	**0.001**
HDP(%)	13/255(5.10)	16/179(8.94)	1.83(0.86–3.91)	0.119	24/150(16.00)	3.55(1.75–7.20)	**<0.001**
Preeclampsia(%)	5/255(1.96)	2/179(1.11)	0.56(0.11–2.95)	0.497	4/150(2.67)	1.37(0.36–5.18)	0.643
Polydydramnios(%)	5/255(1.96)	2/179(1.12)	0.56(0.11–2.91)	0.487	7/150(4.67)	2.45(0.76–7.92)	0.133
Oligodydramnios(%)	14/255(5.49)	13/179(7.26)	1.26(0.57–2.78)	0.568	4/150(2.67)	0.48(0.15–1.49)	0.202
PPPOM(%	2/255(0.78)	3/179(1.68)	1.91(0.31–11.73)	0.484	6/150(4.00)	5.27(1.05–26.55)	**0.044**
PP(%)	5/255(1.96)	4/179(2.23)	1.21(0.32–4.59)	0.781	0.000	N#A	N#A
Placental abruption(%)	1/255(0.39)	1/179(0.56)	1.56(0.10–25.63)	0.755	2/150(1.33)	3.78(0.33–43.37)	0.285
PTB<32weeks(%)	4/255(1.57)	4/179(2.23)	1.48(0.36–5.99)	0.578	3/150(2.00)	1.29(0.28–5.84)	0.743
PTB<37weeks (%)	20/255(7.84)	18/179(10.06)	1.29(0.66–2.51)	0.463	20/150(13.33)	1.80(0.94–3.48)	0.078
CS(%)	182/255(71.37)	144/179(80.45)	1.73(1.04–2.88)	**0.034**	129/150(86.00)	2.44(1.36–4.40)	**0.003**
PPH(%)	0.000	1/179(0.56)	N#A	N#A	2/150(1.33)	N#A	N#A
Fetal macrosomia(%)	30/255(11.76)	33/179(18.44)	1.75(1.02–3.10)	**0.042**	41/150(27.33)	3.07(1.81–5.00)	**<0.001**
Respiratory distress(%)	1/255(0.39)	4/179(2.23)	6.09(0.67–55.53)	0.109	1/150(0.67)	1.56(0.10–25.41)	0.756
SGA(%)	8/255(3.14)	0.000	N#A	N#A	4/150(2.67)	0.90(0.26–3.04)	0.859
LGA(%)	45/255(17.65)	47/179(26.26)	1.67(1.05–2.66)	**0.030**	49/150(32.67)	2.35(1.46–3.74)	**<0.001**
LBW<1,500g(%)	1/255(0.39)	2/179(1.12)	2.81(0.22–35.34)	0.424	3/150(2.00)	3.81(0.35–41.52)	0.272
LBW<2,500g(%)	10/255(3.92)	10/179(5.59)	1.38(0.43–4.44)	0.589	7/150(4.67)	0.67(0.20–2.45)	0.520
GA(w)	39.1(38.3–40.0)	39(38–39.5)	N#A	0.234	39(37.5–39.4)	N#A	**<0.001**
BW(g)	3450(3150–3700)	3600(3200–3800)	N#A	**<0.001**	3500(3180–4000)	N#A	0.075
BH(cm)	50(50–50)	50(50–50)	N#A	0.324	50(50–51)	N#A	0.052
NICU admission(%)	23/255(9.02)	28/179(15.64)	1.81(0.95–3.43	0.071	21/150(14.00)	1.36(0.68–2.71)	0.382
Congenital anomalies(%)	12/255(4.71)	7/179(3.91)	0.84(0.32–2.19)	0.715	10/150(6.67)	1.34(0.56–3.24)	0.510
Mortality(%)	1/255(0.39)	0.000	N#A	N#A	1/150(0.67)	1.43(0.08–26.60)	0.809

### Additional analysis

Tables 7–10 show the comparisons of adverse perinatal outcomes between the target BMI group and BMI reduction group. There was a statistically significant difference for congenital anomalies between the group with a BMI of 30–32 and the group with a BMI of 27–29, representing a 10% reduction in BMI. Apart from congenital anomalies, there was no statistically significant difference regarding other pregnancy outcomes between the two groups. In contrast, women with a BMI of 30–32 were associated with higher risks of GH, fetal macrosomia and LGA when compared with the women with a BMI of 25–27, representing a 15% reduction in BMI. No significant difference was observed regarding the perinatal complications between the group with a BMI of 28–29 and group with a BMI of 25–26, representing approximately a 10% reduction in BMI. Rates of GH, CS and fetal macrosomia were significantly different between the group with a BMI of 28–29 and group with a BMI of 24–25, representing a 15% reduction in BMI. Pregravid BMI in the overweight range was associated with higher rates of GDM, CS, fetal macrosomia, LGA and NICU admission. BMI of 26–27 resulted in increased rates of CS, fetal macrosomia, and LGA when compared with BMI of 23–24, representing a 10% reduction in BMI. The same results were seen between BMI of 26–27 and BMI of 24–25, representing a 5% reduction in BMI. Rates of GDM, LGA and NICU admission were significantly higher among women with a BMI of 24–25 compared with those with a BMI of 21–22, representing a 10% reduction in BMI. Meanwhile, the rates of GDM and NICU admission in women with a BMI of 24–25 were still significantly higher than those in women with a BMI of 22–23, representing a 5% reduction in BMI.

**Table 7:** Comparisons of adverse perinatal outcomes between target BMI group (BMI of 30–32) and BMI reduction group.

	BMI (27–29)		BMI (26–27)	
Parameter	aOR(95%CI)	P value	aOR(95%CI)	P value
GDM	1.29(0.70–2.37)	0.410	1.58(0.90–2.78)	0.113
HDP	1.75(0.98–3.13)	0.058	2.39(1.32–4.30)	**0.004**
Polyhydramnios	1.76(0.69–4.50)	0.238	2.06(0.87–4.88)	0.102
PPROM	1.60(0.51–5.07)	0.423	1.61(0.56–4.64)	0.375
PTB<37w	1.54(0.89–2.68)	0.125	1.91(1.03–3.55)	**0.040**
CS	0.87(0.51–5.07)	0.616	1.06(0.65–1.75)	0.810
Fetal macrosomia	1.18(0.77–1.81)	0.439	1.53(1.02–2.29)	**0.039**
LGA	1.38(0.93–2.02)	0.107	1.64(1.14–2.36)	**0.007**
NRDS	2.37(0.42–13.23)	0.326	2.57(0.56–11.74)	0.223
NICU admission	1.33(0.68–2.54)	0.387	1.20(0.67–2.15)	0.542
Congenital anomalies	2.12(1.05–4.29)	**0.035**	2.39(1.26–4.54)	**0.008**

**Table 8:** Comparisons of adverse perinatal outcomes between target BMI group (BMI of 28–29) and BMI reduction group.

	BMI(25–26)		BMI(24–25)	
Parameter	aOR(95%CI)	P value	aOR(95%CI)	P value
GDM	1.20(0.71–2.05)	0.501	1.09(0.64–1.86)	0.746
HDP	1.70(0.95–3.04)	0.074	2.34(1.27–4.33)	**0.006**
Polyhydramnios	0.87(0.27–2.80)	0.810	1.09(0.32–3.67)	0.835
PPROM	1.07(0.36–3.17)	0.903	0.89(0.31–2.55)	0.375
PTB<37w	0.94(0.55–1.58)	0.800	1.18(0.69–2.01)	0.538
CS	1.22(0.78–1.90)	0.392	1.57(1.02–2.43)	**0.042**
Fetal macrosomia	1.16(0.79–1.70)	0.461	1.43(0.97–2.11)	0.070
LGA	1.13(0.81–1.59)	0.477	1.31(0.93–1.84)	0.121
NRDS	1.39(0.30–6.51)	0.675	1.38(0.29–6.51)	0.683
NICU admission	1.00(0.59–1.69)	0.985	1.11(0.65–1.89)	0.708
Congenital anomalies	1.06(0.52–2.17)	0.868	1.71(0.79–3.68)	0.174

**Table 9:** Comparisons of adverse perinatal outcomes between target BMI group (BMI of 26–27) and BMI reduction group.

	BMI(23–24)		BMI(24–25)	
Parameter	aOR(95%CI)	P value	aOR(95%CI)	P value
GDM	0.98(0.62–1.57)	0.939	1.64(1.06–2.54)	0.766
CS	1.64(1.15–2.34)	**0.001**	1.04(0.79–1.35)	**0.025**
Fetal macrosomia	1.70(1.23–2.34)	0.496	1.05(0.79–1.40)	**0.009**
LGA	1.49(1.12–1.98)	**0.007**	1.11(0.86–1.41)	**0.049**
NICU admission	1.19(0.72–1.96)	**0.001**	1.57(1.03–2.41)	**0.036**

**Table 10:** Comparisons of adverse perinatal outcomes between target BMI group (BMI of 24–25) and BMI reduction group.

	BMI(21–22)		BMI(22–23)	
Parameter	aOR(95%CI)	P value	aOR(95%CI)	P value
GDM	2.26(1.42–3.62)	**0.001**	1.64(1.06–2.54)	**0.025**
CS	1.16(0.89–1.51)	0.260	1.04(0.79–1.35)	0.799
Fetal macrosomia	1.18(0.88–1.58)	0.270	1.05(0.79–1.40)	0.739
LGA	1.34(1.05–1.73)	**0.021**	1.11(0.86–1.41)	0.429
NICU admission	1.51(0.99–2.30)	0.056	1.57(1.03–2.41)	**0.036**

## Discussion

To the best of our knowledge, we, for the first time, evaluated the association between pre-IVF BMI and the risks of negative pregnancy outcomes in Chinese population. Moreover, we assessed the effect of obesity on some particular pregnancy outcomes, such as PPROM and PP, which have not been recognized previously. In the present study, we found that pre-IVF obesity was independently associated with absolute risks of many important obstetric outcomes, including GDM, GH, PE, polyhydramnios, PPROM, placental abruption, PTB<32 weeks, macrosomia, LGA, cesarean delivery rate, NRDS, NICU admission and congenital anomalies. In general, our findings were consistent with previous studies on spontaneous pregnancies [11–17].

The present study reported that pre-IVF obesity was related to a significantly increased risk of polyhydramnios, which has not been well-documented in previous studies. It may occur through the development of GDM. Unexpectedly, the increased risk of polyhydramnios remained significant after adjustment of GDM status, suggesting that female obesity was an independent predictor of polyhydramnios, while its underlying mechanism remained largely unexplored. Maternal obesity seems to be protective from PP, while the potential mechanism remains unclear. According to the present study, PTB<32 weeks and PPH seemed to have a less significant association with BMI in IVF pregnancies. As a matter of fact, there is a notable lack of clarity in the association between BMI and PPH reported in observational studies. However, some studies [18–20] have suggested that maternal obesity is an important risk factor for PPH, while others [21–23] fail to find any effect of BMI on PPH. Several potential explanations can be offered for such conflicting results. On the one hand, different criteria for PPH definition (either blood loss> 500 mL or > 1,000 mL) can be advocated. On the other hand, it is clinically difficult to accurately estimate blood loss, particularly in obstetric scenarios. We observed that obesity was related to PTB<37 weeks, while there was no significant change between obesity and PTB<32 weeks. Such finding was consistent with many previous reports [21–23], while its underlying mechanism remained unknown. In contrast with many previous reports [24–26], we did not find statistically significant difference of neonatal mortality among all BMI groups, even though the obese group exhibited a higher risk. Such discrepancy might be partly attributed to the small sample size and the low number of deaths, and it was also possibly caused by the fact that only severe obesity (BMI≥35 kg/m^2^) had relationship with neonatal mortality.

Evidence suggests that GDM and HDP are associated with adverse outcomes for mother and offspring [27–28]. To assess whether the increased risk of perinatal complications was mediated by development of GDM/HDP, we performed a sensitivity analysis by conducting logistic regressions after adjustment for development of GDM/HDP (in addition to age, parity, PCOS, and type of COH) for those outcomes with a significant association with pre-IVF obesity. The observed increased risks in PPROM, NRDS and congenital anomalies were no longer seen after adjustment of GDM, suggesting that these complications occurred through development of GDM. NRDS is a common complication of GDM, which adversely affects the formation of alveolar surfactants in neonates. Therefore, GDM might be the potential mechanism that obesity potentiated the risk of respiratory distress.

A number of previous studies have reported that there is a statistically significant increase in risks of congenital malformations in offspring of women with pregestational diabetes [29–31], and such risks are increased with degree of maternal hyperglycemia [32]. GDM was also found to be related to congenital malformations in our study. We could not conclude that congenital anomalies occurred through development of GDM, while it might play an important role in the mechanism. Unfortunately, our study was restricted to live births. A part of severe congenital malformations during pregnancies ended in spontaneous miscarriages or stillbirths, which is a process of natural selection. Besides, some malformations could be diagnosed prenatally, leading to induced abortions. Therefore, we might underestimate the magnitude of the problem. When HDP was controlled, NRDS was the only outcome showing a statistically significant change with the increase of BMI. It might be related to the scientific fact that HDP is associated with intrauterine growth restriction and preterm delivery [33].

In patients without PCOS, PPROM and congenital anomalies were not significantly changed with the increase of BMI, although the trends for outcomes were also worsened with the increase of BMI. These results suggested that PCOS was also the underlying pathologies that contributed to the outcomes. PCOS might have underlying metabolic and endocrine influences associated with GDM that contributed to PPROM and congenital anomalies. For patients with PCOS, the risk of PPROM was significantly changed in obese women, further confirming the association between PCOS and PPROM. Apart from PPROM, the risks of GDM, HP, CS, fetal macrosomia and LGA were significantly increased. Further studies are needed to estimate whether there is a synergistic risk of perinatal outcomes in overweight/obese women with PCOS.

The effect of obesity on poor perinatal outcomes has been widely studied. However, the etiology of such influence remains unknown. Recently, emerging novel evidence suggests a potential association among epigenetics, microRNAs (miRNAs) and pregnancy complications [34]. Numerous data have proved that the placenta responds to the maternal obesogenic environment by expressing specific miRNAs. There have been eight miRNAs (miR-100, miR-1269, miR-1285, miR-181, miR-185, miR-214, miR-296 and miR487), which are confirmed to be associated with obesity. Among these miRNAs, five of them (miR-100, miR-181, miR-185, miR-214 and miR-296) are related to type 2 diabetes. Four miRNAs (miR-100, miR-1285, miR-296 and miR-487) are associated with LBW. In addition, miR-296 has been found to be dysregulated in placenta with PE and PTB [35–37]. The dysregulation of placental obesity-associated miRNAs may participate in the mediation of adverse effects of maternal obesity on the offspring. Moreover, Laganà et al. have concluded that several miRNAs are also dysregulated in the sera of women affected by PE, facilitating the miRNA evaluation and thus offering early diagnosis of PE [38]. It is worthwhile to perform large cohort studies to further identify the role of obesity-associated miRNAs to improve early diagnosis and management of the disease.

Most of the studies have focused on the effects of obesity on periantal outcomes and strongly recommended that obese women should take efforts to lose weight pre-conception or pre-IVF. However, weight loss to decrease the risk of poor perinatal complications has been rarely studied. In our present study, we established the weight-loss models to evaluate the effects of weight loss on the risk of poor perinatal outcomes. We found that a 10% reduction in pre-IVF BMI was associated with reduced risk of congenital anomalies for women with a BMI of 30–32. In contrast, larger differences in prepregnancy BMI (15% differences, or more) would be necessary to see meaningfully risk differences for GH, fetal macrosomia and LGA. Our study also found that for women with a BMI of 28–29, a 10% reduction in prepregnancy BMI did not improve the perinantal outcomes. A stricter weight reduction of 15% in pre-IVF BMI might lower the risks of GH, CS and fetal macrosomia. For women with a BMI of 26–27, only a 5% reduction in BMI could significantly reduce the risks of GDM, CS and fetal macrosomia. As to women with a BMI of 24–25, a 5% reduction in pre-IVF BMI might result in reduced rates of GDM and NICU admission. If this target group could fulfill the goal to lose weight with a 10% difference in pre-IVF BMI, they could have their babies at decreased risk of LGA, in addition to reduced rates of GDM and NICU admission.

It is very hard for obese population to lose enough weight to become normal-weight women. Based on this conclusion, a 10%-15% reduction in pregravid BMI was recommended as a weight-loss target to reduce perinatal outcomes for the obese population. In overweight population, just a 5% reduction in pregravid BMI was helpful for the health of both mother and baby, which is such inspiring news for obese people. Therefore, clinicians and patients in China could determine what magnitude of expected reduction in risk was meaningful at an individual level.

The Chinese population is quite different from Western populations in the prevalence of overweight and obesity. Moreover, large differences exist in dietary structure and lifestyle habits. There were only 39 women (39/3,962, 0.98%) with a BMI of 33 or higher in the present study. Therefore, we could not conduct a weight reduction model for the target BMI group with a BMI of 33 or higher. Fortunately, the current prevalence of severe obesity in China is relatively low. Therefore, we do not need to offer the weight-loss goals for the severe obesity group.

This study has several limitations. First, the between-woman differences in prepregnancy BMI were not equal to the same magnitudes of BMI loss for individual women. The lack of controlled trials and sufficient data regarding prepregnancy weight loss, studies that compare the outcomes of different women by prepregnancy BMIs provide clinicians available evidence to offer weight loss counseling. In the absence of data from randomized trials of weight loss interventions, studies that compare the outcomes of different women by prepregnancy BMIs provide clinicians available evidence to offer weight loss counseling. Second, despite a very high response rate, our data on obstetric outcomes were self-reported. Therefore, it might underestimate the magnitude of the problem. Third, our study was restricted to live births. A part of severe congenital malformations during pregnancies ended in spontaneous miscarriages or stillbirths. Besides, some malformations could be diagnosed prenatally, leading to induced abortions. Therefore, we might underestimate the magnitude of association between pre-IVF obesity and congenital anomalies. The last but not the least, although single-center studies had limited size and statistical power, they could also ensure homogeneity in clinical practice.

## Conclusions

Collectively, pregravid obesity served as an independent predictor of adverse birth outcomes in IVF pregnancies. Our results suggested that some risks could occur through development of HDP and GDM. It is hard for obese women to lose enough weight to normal BMI categories. We encouraged obese women to lose weight to a 10–15% reduction in pregravid BMI, which was useful to reduce the risks of some perinatal complications. For overweight women, just a 5% reduction in pregravid BMI was helpful. However, we used BMI definitions of WGOC in the present study, making the recommendations less applicable to general international population. Prospective studies are required to further demonstrate the weight-loss goals to reduce the risks of poor perinatal outcomes for women with high BMI.

### Ethics statement

The study was approved by the institutional review board of the Reproductive Hospital Affiliated to Shandong University. The ethics board approval number is 201424. The data were anonymously analyzed, so no consent was required.

## Supporting information

S1 Data The data underlying the findings are fully available. (XLS)
